# Iterative training set refinement enables reactive molecular dynamics *via* machine learned forces†

**DOI:** 10.1039/c9ra09935b

**Published:** 2020-01-27

**Authors:** Lei Chen, Ivan Sukuba, Michael Probst, Alexander Kaiser

**Affiliations:** Universität Innsbruck, Institut für Ionenphysik und Angewandte Physik 6020 Innsbruck Austria alexander.kaiser@uibk.ac.at; Department of Nuclear Physics and Biophysics, Comenius University SK-84248 Bratislava Slovakia; School of Molecular Science and Engineering, Vidyasirimedhi Institute of Science and Technology Rayong 21210 Thailand

## Abstract

Machine learning approaches have been successfully employed in many fields of computational chemistry and physics. However, atomistic simulations driven by machine-learned forces are still very challenging. Here we show that reactive self-sputtering from a beryllium surface can be simulated using neural network trained forces with an accuracy that rivals or exceeds other approaches. The key in machine learning from density functional theory calculations is a well-balanced and complete training set of energies and forces. We have implemented a refinement protocol that corrects the low extrapolation capabilities of neural networks by iteratively checking and improving the molecular dynamic simulations. The sputtering yield obtained for incident energies below 100 eV agrees perfectly with results from *ab initio* molecular dynamics simulations and compares well with earlier calculations based on pair potentials and bond-order potentials. This approach enables simulation times, sizes and statistics similar to what is accessible by conventional force fields and reaching beyond what is possible with direct *ab initio* molecular dynamics. We observed that a potential fitted to one surface, Be(0001), has to be augmented with training data for another surface, Be(011̄0), in order to be used for both.

## Abstract 2

Reactive self-sputtering from a Be surface is simulated using neural network trained forces with high accuracy. The key in machine learning from DFT calculations is a well-balanced and complete training set of energies and forces obtained by iterative refinement.

## Graphical abstract

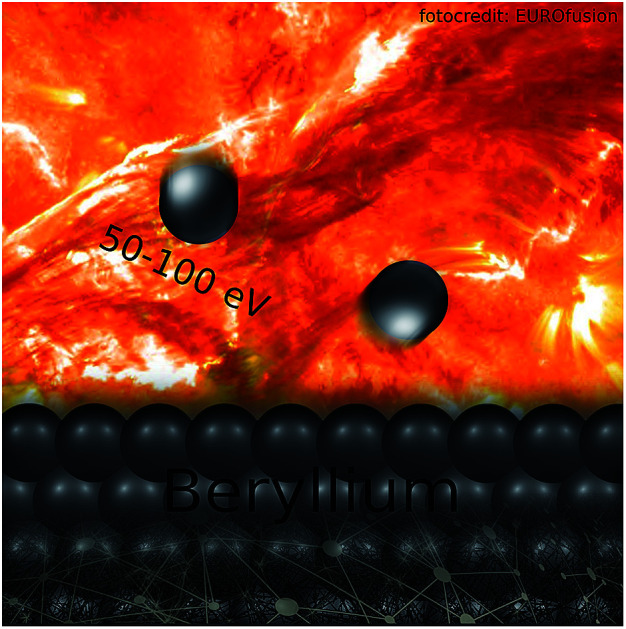

## Introduction

In molecular dynamics (MD) simulations energies and forces are complicated functions of nuclear coordinates and element types. Calculating forces on-the-fly by electronic structure methods avoids having to handle these functions explicitly but is, even with density functional methods, still restricted to small systems and short times, compared to MD with analytic potential energy functions. Even for non-reactive systems the development of a reliable force field is very tedious. Consequently, machine learning approaches are developed to fill this gap by learning energy and forces from quantum chemical data and to replace a conventional force field.^1–5^ Feedforward Neural Networks^4,6–8^ and Gaussian Approximation Potentials^9–12^ are most widely used at present. In both of them Cartesian coordinates of the atoms are first transformed into symmetry invariant atom-centered representations by various methods.^7,13^ The present work applies feedforward neural networks and the Behler–Parrinello type^6^ atomistic representation. The parameters are the bias parameters that act on individual neurons and the weights that interconnect artificial neurons in different layers. The number of parameters only depends on the size of the neural network. When increasing the number of parameters, the network can basically store the information it is trained on almost perfectly and it can also interpolate to some extent.

We can train the network on a finite set of energies and forces and its quality will depend very much on the choice and size of the training set and on the power of the global optimizer to reach a low-lying minimum. Compared to the huge positional phase space spanned by all combinations of atomic positions, the number of configurations (∼6000 in this work) used to train the network is meagre.

In this work, we implement a refining procedure based on previous work^4,14^ for training set construction and we train a neural network potential (NNP) for molecular dynamics simulations of reactive beryllium (Be) self-sputtering and show that our NNP based simulations are accurate in predicting sputtering yields. Knowledge of the stability of Be surfaces is very relevant because beryllium sheets have been chosen as armor material in the first wall of the ITER reactor currently being constructed.^15^ Having only 4 valence electrons, beryllium has the additional advantage that it can be treated with density functional calculations rather efficiently, making comparisons with *ab initio* MD feasible. Previous work on this system include an MD study by Ueda *et al.* where a pair potential was developed and self-sputtering processes of Be at low incident energies (≤100 eV) was simulated.^16^ Björkas *et al.* developed a bond order potential for the ternary system Be–C–H, and the Be potential was applied to MD simulations of Be self-sputtering.^17^

## Training set generation and refinement

**Fig. 1:**
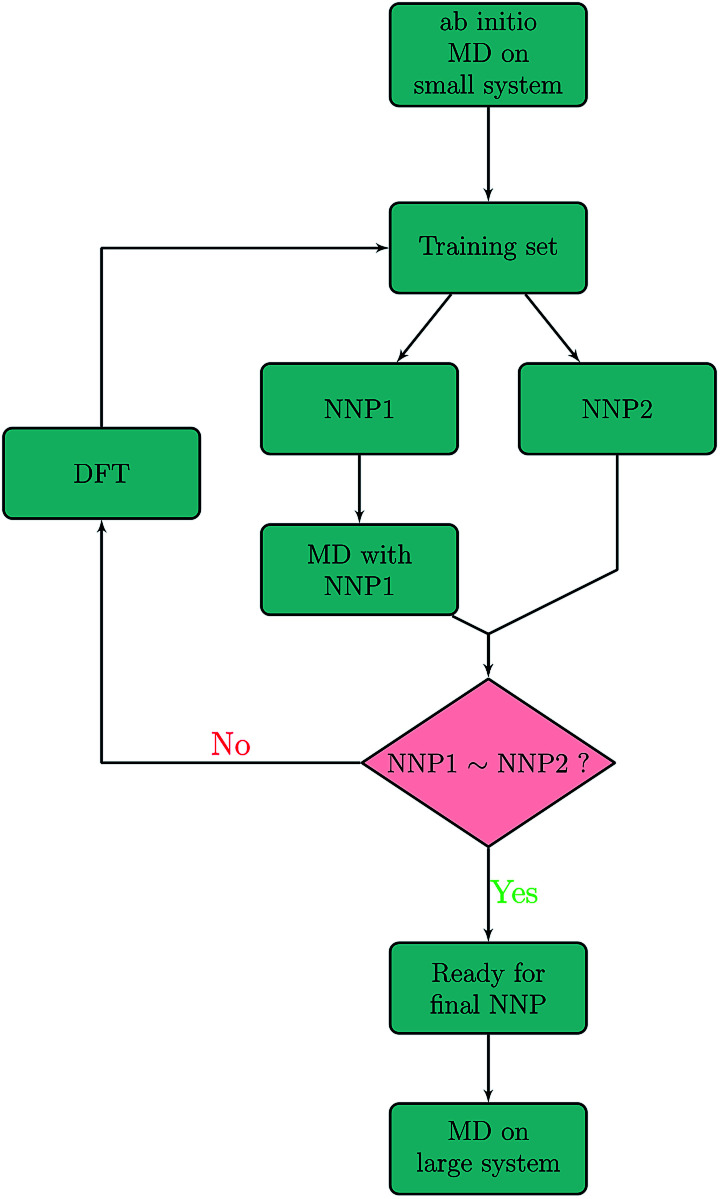
Schematic of the iterative refinement procedure based on previous work^4,14^ for training set generation. Energies and forces predicted with NNP1 and NNP2 are compared with each other along molecular dynamics trajectories generated with NNP1. The decision (NNP1∼NNP2?) of including a particular phase-space point into the training set is made either by the energy criterion |*E*(NNP1) − *E*(NNP2)| > *ε*_E_ or the force criterion |*F*_*x*,*y*,*z*_(NNP1) − *F*_*x*,*y*,*z*_(NNP2)| > *ε*_F_.

The NNP is trained on energies and forces obtained with plane-wave DFT calculations. Details of the DFT and *ab initio* MD calculations are given in the section on Computational methods. Artrith and Behler have already described a refinement procedure based on dynamic simulations to extend the accuracy and applicability of a neural network potential for MD simulations.^4,14^ In the present work, our refining procedure also relies on the assumption that two different neural networks that have been fitted to the same data set will deliver approximately the same result for well-sampled regions of the phase space but not in extrapolations out of these regions. This allows to systematically and automatically identify structures that are missing in the training data. The iterative procedure is schematically shown in Fig. 1. In a first step, configurations were created by randomly extracting snapshots from 500 *ab initio* MD sputtering trajectories on a small Be(0001) surface slab with 96 atoms. Two preliminary NNPs, NNP1 and NNP2 were fitted to this training set. NNP1 and NNP2 have the same topology and differ only in the starting values of their fit parameters which are randomly chosen. They are simple feedforward 53 × 30 × 30 × 1 NNs with two hidden layers. We found that including more parameters or making the neural network deeper does not improve the accuracy any more. More details on the employed symmetry functions and the neural network are given in the Computational methods.

**Fig. 2:**
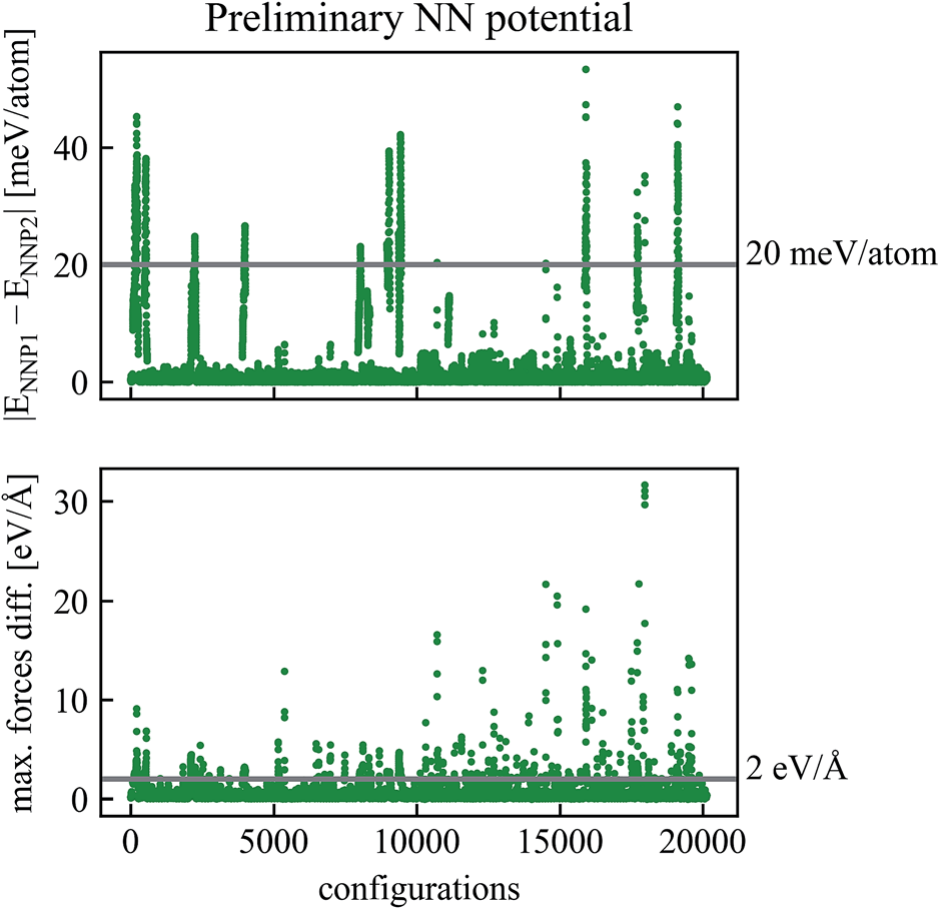
Comparison of energies and forces of the preliminary NN potentials NNP1 and NNP2 along the same trajectories. Configurations with absolute values of energy differences higher than *ε*_E_ = 20 meV per atom or maximum force differences higher than *ε*_F_ = 2 eV Å^−1^ have been recalculated by DFT and added to the training data to obtain the refined NNP in Fig. 3.

The refinement procedure starts with using NNP1 for short MD simulations (40 fs) of the Be self-sputtering process at various impact energies on the surface slab with 96 atoms. The energies and forces of these new configurations were then predicted by NNP2 along the same trajectories, and energies and forces of both networks were compared with each other as shown in Fig. 2. Configurations with energy differences larger than *ε*_E_ = 20 meV per atom or maximum force differences larger than *ε*_F_ = 2 eV Å^−1^ were selected and subjected to a DFT calculation of energies and forces which were then added to the training data.

**Fig. 3:**
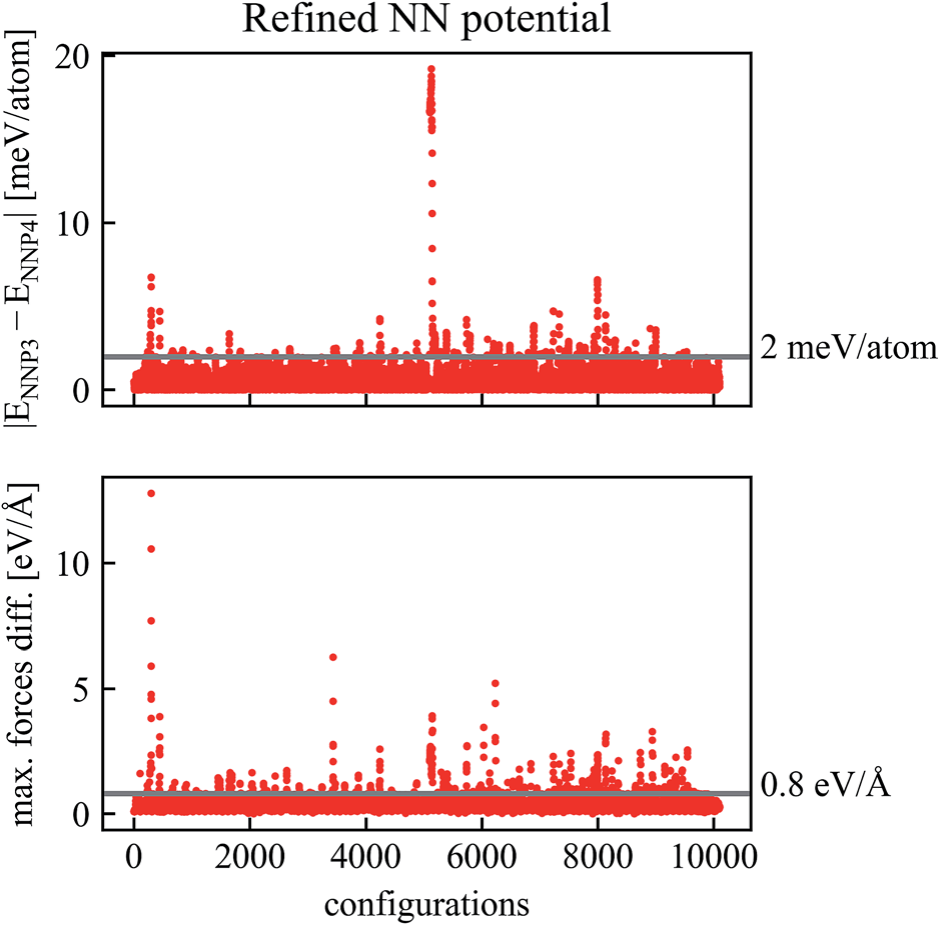
Comparison of energies and forces of the refined NN potentials NNP3 and NNP4 along the same trajectories. Configurations with absolute values of energy differences higher than *ε*_E_ = 2 meV per atom or force differences higher than *ε*_F_ = 0.8 eV Å^−1^ have been recalculated by DFT and added to the training data to obtain a final NNP.

Two new neural network potentials NNP3 and NNP4 were fitted to the refined training set and their differences for new trajectories is shown in Fig. 3. It is apparent that already after one cycle of the refinement process, the differences between the two NNPs decrease considerably. For most configurations in Fig. 3, the energy and maximum forces differences between NNP3 and NNP4 are within 5 meV per atom and 1 eV Å^−1^. Due to this excellent improvement, we reduced the number of trajectory calculations in the second refinement step.

Further refinement can be done iteratively as indicated in Fig. 1. In our case, a second refinement step with much smaller energy and force thresholds (*ε*_E_ = 2 meV per atom, *ε*_F_ = 0.8 eV Å^−1^) was sufficient.

With this iterative refinement process, the final reference data set consists of 5871 configurations containing 97 atoms each. Thus, 5270 energies and 1 533 486 forces are used to fit the final potential energy function NNP5 and 601 energies and 174 870 forces are part of the test set which is not used for training but rather to validate the potential and to prevent overfitting. After 60 training epochs, the root mean square errors (RMSE) in the test set converged to 0.7 meV per atom for energies and 32.0 meV Å^−1^ per atom for forces, very close to the corresponding value in the training set (0.6 meV per atom and 31.8 meV Å^−1^).

## Static performance of the refined neural network potential

**Fig. 4:**
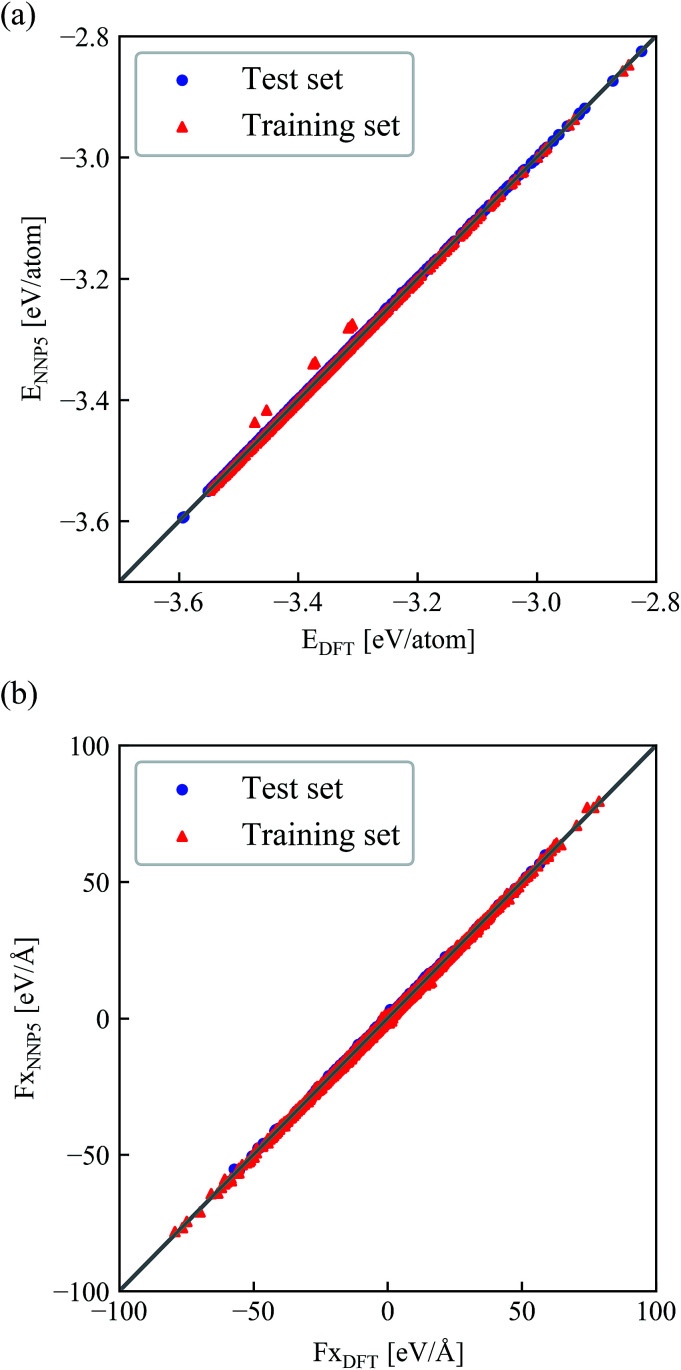
Correlation between the NNP5 and DFT energies per atom (a) and *x*-components of the forces (b) for all configurations in the training and test set.

The correlation of NNP5 and DFT energies and forces for each atom are shown in Fig. 4(a) and (b). Only the *x*-component of the forces is shown here since *y* and *z* have been inspected but give virtually identical plots. DFT and NNP5 energies are very close except for very few configurations in the training set. Similarly, the values of NNP5 and DFT forces at all three directions are perfectly correlated.

We are now in a position to go to a larger system, a Be(0001) surface with 490 atoms. This surface will later be used for the sputtering simulations. The equilibrium lattice constants of using NNP5 turn out reasonable (Table 1) with NNP5 and DFT showing relative differences of less than 2% compared to experimental values. The NNP5 total energy for this configuration is also very close to the DFT value with an absolute deviation of 3.3 meV per atom. Relaxation of the surface to its equilibrium configuration is necessary for the subsequent sputtering calculations since otherwise, a large amount of potential energy is heating the system at the beginning that could irreversibly change the structure due to expansion and melting.

**Table 1:** Lattice constants and total energy of the Be(0001) surface from NNP5 and DFT calculations. Both simulations used a periodic slab consisting of 490 atoms

Lattice constants (Å)			Total energy (eV)		Energy difference per atom (meV per atom)
NNP5	DFT	Exp.^18^	*E*_NNP5_	*E*_DFT_	(*E*_NNP5_ − *E*_DFT_) per atom
*a* = 2.28; *c* = 3.55	*a* = 2.28; *c* = 3.54	*a* = 2.29; *c* = 3.58	−1791.7	−1790.1	−3.3

## Performance of the refined neural network potential in reactive sputtering simulations

**Fig. 5:**
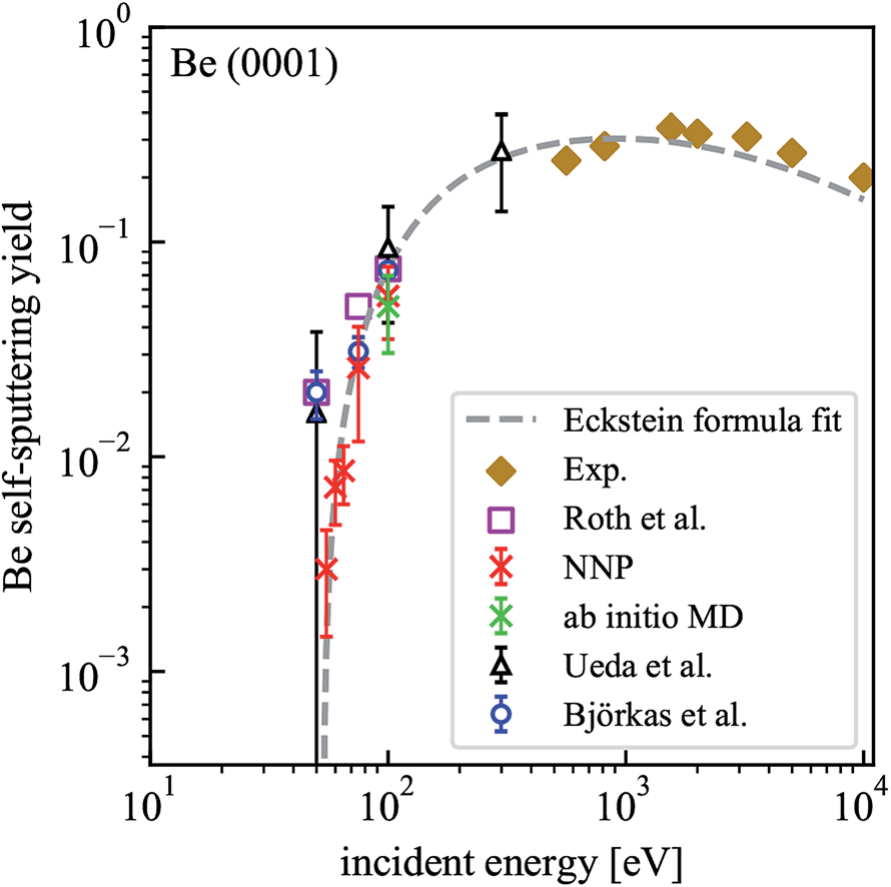
Dependence of the self-sputtering yield for the Be(0001) surface on the incident energy. The results of MD simulations by Ueda *et al.*^16^ and Björkas *et al.*,^17^ the data of Monte Carlo simulations (assuming a surface binding energy of 3.38 eV) by Roth *et al.*^21^ and experimental results^22,23^ are included for comparison.

Although only a small system was used to train NNP5, the energy contribution from each atom depends only on the local chemical environment and therefore it can be used to simulate a larger system. MD simulations performed on the Be(0001) surface slab with 490 atoms result in a self-sputtering yield of 5.6% which agrees perfectly with a yield of 5% obtained from 500 *ab initio* MD trajectories calculated on the same system under identical conditions for 100 eV incident energy. The sputtering yields of all our simulations are summarized in Table 2 and compared with other MD simulations, Monte Carlo data and experimental estimates in Fig. 5. Statistically, their accuracy increases with the number of simulation runs and the error bars are estimated by 
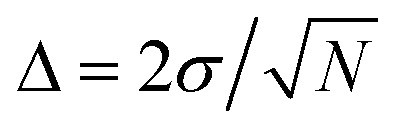
 where the standard deviation *σ* is obtained by assuming a Bernoulli distribution of *N* trajectories. The NNP5 sputtering yields fall well within other available data. Timings included in Table 2 show that the NNP5-based MD simulations are more than two orders of magnitude faster than their *ab initio* MD simulations.

**Table 2:** Sputtering yields and statistical error estimates for the Be(0001) surface obtained from neural network based MD simulation at various incident energies and from an *ab initio* MD simulation at 100 eV incident energy. The average computational time for each NNP based MD and *ab initio* MD simulation is compared. Note that we have performed 5000 trajectories for low incident energies (55 eV, 60 eV and 65 eV) using NNP5 to obtain a lower error bar

Crystal size (Å)		Sputtering yield of Be(0001)						CPU time/trajectory
		50 eV	55 eV	60 eV	65 eV	75 eV	100 eV	
15.6 × 15.6 × 30.1 (490 atoms)	NNP5	0	0.003 (0.0015)	0.0072 (0.0024)	0.0086 (0.0026)	0.026 (0.014)	0.056 (0.021)	10 minutes (4 cores)
	*ab initio* MD	—	—	—	—	—	0.050 (0.019)	30 hours (16 cores)
22.8 × 22.8 × 48.4 (2000 atoms)	NNP5	—	—	—	—	—	0.054 (0.020)	

For all incident energies, we never encountered reflections of the Be projectile. In our energy range this is expected and agrees with the simulation results from Ueda *et al.*^16^ where a pair potential had been used. No atoms were sputtered for incident energies below 50 eV, which is consistent with the findings from Ueda *et al.* only if the large error bars are taken into account but not in good agreement with the BOP-based results of Björkas *et al.* who reported a similar value than Ueda *et al.* at 50 eV incident energy but with a much smaller statistical error.^17^ The sputtering threshold energy defined by no sputtering event occurring in 500 trajectories lies within 50 and 55 eV for our setup with NNP5 and is higher than previous estimates from 16–25 eV.^17,19^

We used an estimated sputtering threshold energy *E*_th_ = 53 eV, the parameters *q* = 0.82, *μ* = 1.34 and *λ* = 2.03 from literature^19^ and the experimental data at high energies to fit the sputtering yields to the Eckstein formula.^20^ The resulting function is also included in Fig. 5. Since the available experimental data is in the keV range, it is not possible to make a direct comparison with our simulated results. At an incident energy of 100 eV, our result is very close to the values from Roth *et al.*,^21^ Ueda *et al.*^16^ and Björkas *et al.*^17^ At 75 eV, the NNP5 based sputtering yield is very close to that from the bond-order potential.^17^

In order to check the convergence of our model system with respect to surface size, we also simulated a system with 2000 atoms (the crystal size is given in Table 2) at an incident energy of 100 eV. With 500 simulation runs, we obtained 27 sputtering events (sputtering yield 0.054) and obtained very good agreement with the smaller system (sputtering yield 0.056).

## Transferability of the neural network potential

**Fig. 6:**
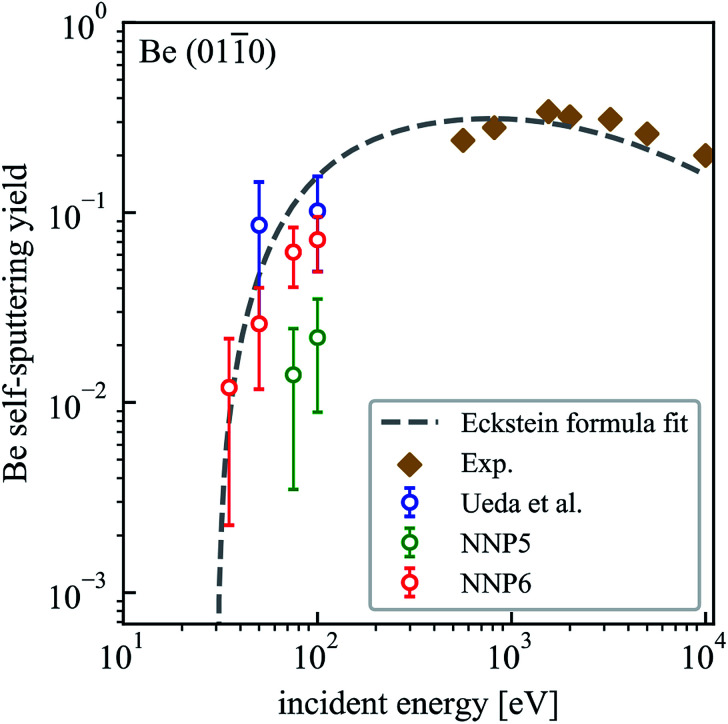
Dependence of the self-sputtering yield of the Be(011̄0) surface on the incident energy. The results of MD simulations by Ueda *et al.*^16^ and experimental results^22,23^ are included for comparison.

With the purpose of testing the transferability of NNP5 to a different surface structure that had not been included in the training set, we performed self-sputtering simulations with an incident energy of 75 and 100 eV on a Be(011̄0) surface consisting of 480 atoms. We obtained much smaller sputtering yields than reported by Ueda.^16^ Applying an iterative refinement step as described above on Be(011̄0) and refitting the neural network, more reasonable results are obtained, albeit of course now with a different potential (NNP6). The sputtering yields for the Be(011̄0) surface using NNP6 are summarized in Table 3 and plotted in Fig. 6. A fit to the Eckstein formula is also shown. We used the same values of the parameters *q*, *μ* and *λ* as for the Be(0001) surface but a lower estimate of the threshold energy *E*_th_ = 30 eV.^20^ The sputtering yield at 100 eV for NNP6 is much larger than the one from NNP5 and is comparable to Ueda's results.^16^ The sputtering yields for the (0001) and (011̄0) surfaces are close to each other at 100 eV, while at lower energies the (011̄0) surface is more susceptible to sputtering with the simple reason that the Be(0001) surface is more stable. In fact, the DFT calculated surface binding energy of 5.13 eV for the (0001) surfaces is much higher than 2.48 eV for the (011̄0) surface.^24^ Finally, we note that the upgrade from NNP5 to NNP6 conserves the accuracy for the (0001) surface with a sputtering yield of 0.052 (0.056) for NNP6 (NNP5) on 500 trajectories.

**Table 3:** Calculated sputtering yields and statistical error estimates for the Be(011̄0) surface obtained from neural network based MD simulations at various incident energies

Crystal size (Å)		Sputtering yield of Be(011̄0)				
		20 eV	35 eV	50 eV	75 eV	100 eV
13.7 × 17.7 × 28.8	NNP5			—	0.014 (0.010)	0.022 (0.013)
	NNP6	0	0.012 (0.010)	0.026 (0.014)	0.062 (0.021)	0.072 (0.023)
—	Ueda *et al.*^16^	—	—	0.086 (0.059)	—	0.102 (0.053)

## Summary

The self-sputtering simulations based on the refined neural network potential give promising results for small and large periodic cell-sizes of the Be(0001) surface. The sputtering yields agree with full *ab initio* Born–Oppenheimer MD results at 100 eV incident energy. Compared to literature data, our simulation protocol seems to be quite competitive while not requiring manual potential development. Transferability to another surface direction was only possible by additional refinement and including data of the new surface into the training data. Although the neural network can only be used for scenarios that are included in the training data and this involves thousands of *ab initio* single-point calculations, the final neural network potential can be used on longer time-scales and larger systems. Especially, there is no difference conceptually in treating more complicated systems such as alloys, where the construction of conventional force fields becomes increasingly cumbersome. As a next step, we plan to apply such simulations to other plasma–wall interactions, in particular considering the ternary H–Be–W system.

## Computational methods

### Neural network potential

In this work, we train a neural network potential of the Behler–Parrinello type.^6^ The neural network code *n*2*p*2 (ref. 25 and 26) recently developed by A. Singraber *et al.* is based on Behler's work.^6,7^ It includes both force and energy fitting and implements a Kalman filter optimizer^26^ which can deal with the large number of data points when forces are included in the fitting. It has also been linked to the versatile molecular dynamics code LAMMPS that we used to integrate the sputtering trajectories.^27,28^ In the Behler–Parrinello approach,^6^ the total energy *E*^pot^ of one configuration is the sum of atomic energies *E*_i_ provided by element-specific neural networks that depend on the local atomic neighbourhood only. Atomic coordinates are transformed to symmetry-invariant atom-centered symmetry functions before entering the neural network in the input nodes. Efficient fitting to forces requires analytic gradients implemented in the *n*2*p*2 library. We chose a simple feedforward neural network topology with two hidden layers with 30 neurons each. The so-called soft-plus activation function^29,30^ which is a smooth approximation of rectified linear units (RELU)^29^ was used as recommended.^31^ A cutoff radius of 7 Å is sufficient to include all relevant neighbour atoms.^32^ The input consists of 9 radial, 24 angular narrow and 20 angular wide Behler-type symmetry functions^7,25^ as detailed in the ESI.†

### Density functional theory

The static DFT and the *ab initio* MD simulations to generate training data were carried out using the Vienna *Ab initio* Simulation Package (VASP).^33,34^ The core and valence electrons were described by the Projector Augmented Wave (PAW)^35^ method and the Perdew–Burke–Ernzerhof (PBE)^36^ exchange–correlation functional. A plane wave basis set with a cut-off energy of 350 eV with periodic boundary conditions was used. The PAW potential for beryllium was used as provided in the VASP library. A Gamma-centered *k*-point mesh of 3 × 3 × 3 was employed. The initial training set was generated by performing Born–Oppenheimer *ab initio* MD on a small hexagonal closely packed Be surface (0001) with 96 atoms (9.1 × 9.1 × 20.6 Å). It was first relaxed with a convergence criterion of 10^−5^ eV on the total energy (about 10^−4^ meV per atom) and of 1 meV Å^−1^ on the forces. Subsequently, the relaxed surface was equilibrated at 300 K for 2 ps within the canonical ensemble using the Nosé–Hoover algorithm.^37,38^ Then perpendicular impacts of single Be atoms with energies of 20, 35, 50, 75 and 100 eV starting from a distance of 5 Å above the surface were simulated. 100 *ab initio* MD runs were performed for each impact energy. The time step was chosen to be 0.5 fs and one run lasted 150 fs for low impact energies (20, 35, 50 eV). Impacts with energies of 75 and 100 eV were simulated for 50 fs.

### MD simulations of sputtering on neural network potentials

In our MD simulations of non-accumulative self-sputtering, an incident neutral Be atom impacts on a pristine Be surface. The target consists of 490 atoms with a size of 15.6 × 15.6 × 30.1 Å. Its crystal structure was relaxed and equilibrated for 2 ps at 300 K within the NVT ensemble using the Nosé–Hoover thermostat^37,38^ before running the trajectories. The incident particle was initially placed 5 Å above the surface while its *x* and *y*-coordinates were randomly chosen. Kinetic energies of 50, 55, 60, 65, 75, and 100 eV were assigned to it by a respective initial velocity in *z*-direction, thus only impacts perpendicular to the surface were simulated in this work. Trajectories were initially integrated for 120 fs with an integration step of 0.2 fs. Further 120 fs integration time were added to those trajectories where the decision of an observed sputtering event could not be made after the first 120 fs. 500 separate MD runs were performed for each incident energy. For the MD simulations in the training data refinement process, the computational details are identical to the sputtering simulations for larger surface systems except for a shorter integration time of 40 fs.

## Conflicts of interest

There are no conflicts to declare.

## Supplementary Material

RA-010-C9RA09935B-s001
