# Supplementary material for: Iterative training set refinement enables reactive molecular dynamics via machine learned forces
Source: RSC Adv. 2020 Jan 27;10(8):4293–9. doi: 10.1039/c9ra09935b (PMC9049032; doi:10.1039/c9ra09935b)
Supplement: RA-010-C9RA09935B-s001 [file RA-010-C9RA09935B-s001.pdf]

Electronic Supplementary Material (ESI) for RSC Advances.
This journal is © The Royal Society of Chemistry 2020

Supplementary Information

Iterative training set refinement enables reactive molecular dynamics via machine learned forces

Lei Chen,[a] Ivan Sukuba,[a,b] Michael Probst,[a] and Alexander Kaiser*[a]

[a]Universität Innsbruck, Institut für Ionenphysik und Angewandte Physik, 6020 Innsbruck, Austria
[b]Department of Nuclear Physics and Biophysics, Comenius University, SK-84248 Bratislava, Slovakia
*Electronic mail: alexander.kaiser@uibk.ac.at

Radial, angular narrow and angular wide symmetry functions are defined as (1-3) respectively that are originally from Ref.[1-3].

$$G_i^{rad} = \sum_{j \neq i} e^{-\eta(R_{ij} - R_s)^2} f_c(R_{ij}) \tag{1}$$

$$G_i^{ang.n.} = 2^{1-\zeta} \sum_{\substack{j,k \neq i \\ j<k}}^{all} (1 + \lambda \cos\theta_{ijk})^{\zeta} e^{-\eta(R_{ij}^2 + R_{ik}^2 + R_{jk}^2)} f_c(R_{ij}) f_c(R_{ik}) f_c(R_{jk}) \tag{2}$$

$$G_i^{ang.w.} = 2^{1-\zeta} \sum_{\substack{j,k \neq i \\ j<k}}^{all} (1 + \lambda \cos\theta_{ijk})^{\zeta} e^{-\eta(R_{ij}^2 + R_{ik}^2)} f_c(R_{ij}) f_c(R_{ik}) \tag{3}$$

Table S1 Parameters of symmetry functions employed to describe the local atomic environments in the input layer of neural network. R_c is the cutoff radius and the meaning of the rest parameters refer to the definitions in articles.[1-3]

Symmetry functions of type G_i^{rad}			
No.	η (Bohr^{-2})	R_s (Bohr)	R_c (Bohr)
1	1.3	2.0	13.0
2	1.0	3.0	13.0
3	0.7	3.8	13.0
4	0.7	4.6	13.0
5	0.7	5.4	13.0
6	0.7	6.2	13.0
7	0.7	7.0	13.0
8	0.7	8.5	13.0
9	0.7	10.0	13.0

Symmetry functions of type $G_i^{ang.n}$				
No.	η (Bohr^{-2})	λ	ζ	R_c(Bohr)
1	0.2283	-1	1.0	13.0

2	0.2283	1	1.0	13.0
3	0.2283	-1	4.0	13.0
4	0.2283	1	4.0	13.0
5	0.2283	-1	9.0	13.0
6	0.2283	1	9.0	13.0
7	0.2283	-1	1.0	13.0
8	0.2283	1	1.0	13.0
9	0.2283	-1	4.0	13.0
10	0.2283	1	4.0	13.0
11	0.2283	-1	9.0	13.0
12	0.2283	1	9.0	13.0
13	0.2283	-1	1.0	13.0
14	0.2283	1	1.0	13.0
15	0.2283	-1	4.0	13.0
16	0.2283	1	4.0	13.0
17	0.2283	-1	9.0	13.0
18	0.2283	1	9.0	13.0
19	0.2283	-1	1.0	13.0
20	0.2283	1	1.0	13.0
21	0.2283	-1	4.0	13.0
22	0.2283	1	4.0	13.0
23	0.2283	-1	9.0	13.0
24	0.2283	1	9.0	13.0

Symmetry functions of type $G_i^{ang.w}$

No.	η (Bohr^{-2})	λ	ζ	R_c(Bohr)
1	0.7	-1	1.0	13.0
2	0.7	1	1.0	13.0
3	0.7	-1	6.0	13.0
4	0.7	1	6.0	13.0
5	0.7	-1	1.0	13.0
6	0.7	1	1.0	13.0
7	0.7	-1	6.0	13.0
8	0.7	1	6.0	13.0
9	0.7	-1	1.0	13.0
10	0.7	1	1.0	13.0
11	0.7	-1	6.0	13.0
12	0.7	1	6.0	13.0
13	0.7	-1	1.0	13.0
14	0.7	1	1.0	13.0
15	0.7	-1	6.0	13.0
16	0.7	1	6.0	13.0
17	0.7	-1	1.0	13.0
18	0.7	1	1.0	13.0
19	0.7	-1	6.0	13.0
20	0.7	1	6.0	13.0

1. J. Behler and M. Parrinello, *Phys. Rev. Lett.*, 2007, **98**, 146401.
2. J. Behler, *J. Chem. Phys.*, 2011, **134**, 074106.

3. A. Singraber, J. Behler and C. Dellago, *J. Chem. Theory. Comput.*, 2019, **15**, 1827-1840.
